# Endophytic fungi of *Aegle marmelos* as a source of novel antibacterials and anti-SARS-CoV agents

**DOI:** 10.1140/epje/s10189-026-00565-z

**Published:** 2026-03-11

**Authors:** Soniya Goyal, Poonam Bansal, Pardeep Kumar, Dinesh Kumar, Gunjan Sharma, Ashish Sharma, Bhupesh Gupta, Mahiti Gupta, Raman Kumar, Ahmad Umar, Tubia Almas, Sotirios Baskoutas

**Affiliations:** 1 Department of Bio-Sciences and Technology, Maharishi Markandeshwar (Deemed to Be University), 133207, Mullana, Haryana India; 2 Department of Mechanical Engineering, Maharishi Markandeshwar (Deemed to Be University), 133207, Mullana, Haryana India; 3 Department of Biotechnology, Gujarat Biotechnology University, GIFT City, Gandhinagar, 382355 Gujarat India; 4 Department of Botany and Environment Studies, DAV University, Jalandhar, 144012 Punjab India; 5 Department of Computer Science and Engineering, Maharishi Markandeshwar (Deemed to Be University), 133207, Mullana, Haryana India; 6 Department of Chemistry, Faculty of Science and Arts, and Promising Centre for Sensors and Electronic Devices (PCSED), Najran University, Najran-11001, Kingdom of Saudi Arabia; 7 STEM Pioneers Training Lab, Najran University, Najran-11001, Kingdom of Saudi Arabia; 8 Department of Materials Science and Engineering, The Ohio State University, Columbus, OH 43210 USA; 9 Department of Materials Science, University of Patras, 26504 Patras, Greece

## Abstract

Drug resistance in microorganisms is a growing global threat, highlighting the urgent need for novel therapeutic agents. This study evaluates the antibacterial and anti-SARS-CoV potential of fungal endophytes isolated from *A. marmelos*. Among 16 endophytes screened, the ethyl acetate extract of the #3 AMLBF strain (*Fusarium vanettenii*) exhibited the strongest antibacterial activity against several pathogens. This extract showed minimum inhibitory concentrations (MICs) ranging from 0.49 to 0.8 µg/ml. GC/MS analysis of the active extract identified 45 compounds. Furthermore, molecular docking against the SARS-CoV-2 spike glycoprotein revealed nine potential ligands, with Anthraergosta-5,7,9,22-tetren-3-ol *p*-chlorobenzoate showing the most potent binding affinity (− 10.2 kcal/mol), a value exceeding that of the standard chloroquine (− 6.5 kcal/mol). These results indicate that the organic extract from this fungal strain is a promising candidate for the discovery of new antimicrobial agents.

**Graphical abstract:**

**Figure:**
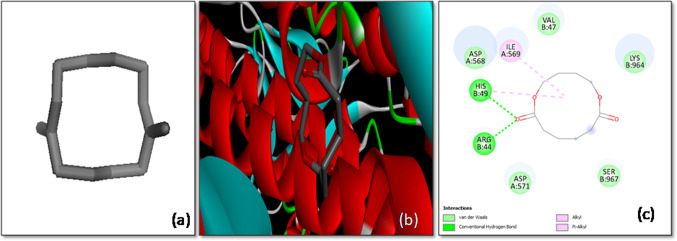
Potential of endophytic fungi

## Introduction

Endophytes are polyphyletic, ubiquitous microorganisms that asymptomatically colonize healthy and mature tissues of host plant. These endophytes can invade plants through lateral invasion or horizontally through soil [1]. Endophytes generally have two phases in their life cycle one above the ground within the plant tissues and other below the ground in soil [1]. Residing in either of them, these endophytes remain in latent state producing some secondary metabolites that mutually benefit each other. They help plants to overcome biotic and abiotic unfavorable conditions by secreting siderophores, calcium and phosphorous solubilizers, and indole acetic acid [2, 3]. Hence, during this symbiotic relationship these endophytes start producing similar biomolecules as their host plant thereby mimicking some of its host’s properties [3, 4]. Once considered as waste products, these secondary metabolites are now considered as life savers. Researchers have increasingly recognized the value of Ayurvedic compounds, leading to the isolation of approximately 100,000 different secondary metabolites, with medicinal plants and their associated endophytes being major contributors [2]. Among these, endophytic fungi have gained attention for their ability to produce a wide range of bioactive compounds with significant antimicrobial, anticancer, antidiabetic, antiobesity, and immunomodulatory properties. Several bioactive compounds such as taxol and camptothecin are in advanced stages of clinical trials.

The promising result of any antimicrobial agent is jeopardized by the resistance shown to that drug by the target microbe. Drug resistance has been recognized about 64 years ago when *E.coli* broke the cycle of penicillin by producing penicillinase. Twenty-first century had witnessed many new types of infectious agents and their resistance to leading drugs. During COVID-19, many patients complain of bacterial and fungal infections which were cured by prescribed drugs without future interventions hence leading to drug resistance [3]. The changes in biochemical and physiological metabolism of existing microbes lead to drug resistance [4]. Scientists are constantly working to break this cycle of drug resistance by targeting other enzymes or sites in microbial metabolism using new pharmacophores [5]. Now we have moved back to era of safer traditional medicines to deal with this ghastly problem. Metabolites produced by microorganisms are the treasure for the scientists in search of new antimicrobial drugs. Endophytes have always been donor of bioactive compounds that are quite useful for humans. Many antimicrobial compounds like podophyllotoxin, hypericin, tanshinone I are gifts of endophytic fungi successfully being used for treating target microbes [5]. Vancomycin has proved to be a critical treatment against Methicillin-resistant *Staphylococcus aureus* [6]. Thus, the secondary metabolites produced by endophytic fungi represent a valuable resource with significant potential for future therapeutic advancements.

The COVID-19 pandemic, caused by the respiratory syndrome coronavirus 2 (SARS-CoV-2) virus, emerged as a major global health crisis in late 2019, starting in Wuhan, China, and swiftly spreading worldwide throughout 2020 [7]. This pandemic eventually leads to widespread illness, death, and significant societal and economic disruptions across the world. The impact of COVID-19 varied widely, ranging from asymptomatic cases to severe respiratory conditions, acute respiratory distress syndrome (ARDS), and, in some instances, death [7]. These risk factors varied with older age, cardiovascular diseases, diabetes, respiratory diseases, and compromised immune systems.

The infection process of SARS-CoV-2 begins with the virus attaching to host cells. The spike (S) protein on the surface of SARS-CoV-2 binds to the angiotensin-converting enzyme 2 (ACE-2) receptor on the host cell surface [8, 9]. The key structural proteins of virus are spike (S), envelope (E), membrane (M), and nucleocapsid (N) and play crucial roles in its virulence, facilitating replication, transmission, and interaction with host cells [10]. The *S*-glycoprotein, which consists of two subunits (S1 and S2), assists the virus in entering the host cell by interacting with the ACE-2 receptor. Targeting the *S*-glycoprotein represents a promising and novel approach for COVID-19 treatment [11].

As per WHO reports, several COVID-19 medications such as lopinavir, ritonavir, chloroquine, hydroxychloroquine, and remdesivir were authorized for emergency use and administered globally to combat the spread of SARS-CoV-2 virus [12]. In patients with comorbidities and immunocompromised, these drugs did not have better efficacy [9]. Hence, ongoing research focuses on understanding virus, its variants, and developing novel strategies to combat with COVID-19. Preventing the attachment of viral particles to host cells could be a highly effective strategy for treating COVID-19. To manage and combat the COVID-19 pandemic effectively, it is crucial to understand the structure, behavior, and transmission mechanisms of SARS-CoV-2. Further research was carried out to explore the potential use of natural products in the treatment or management of COVID-19 [13]. Natural products including plant-based compounds or of microbial origin have been a subject of interest due to their medicinal properties such as potential antiviral activities [14]. In this study, we aim to investigate the antiviral potential of secondary metabolites derived from the endophytic isolate #3AMBLF against SARS-CoV-2 through a molecular docking approach.

## Material and methods

### Isolation of endophytic fungi

Fungal endophytes were isolated from *A. marmelos* (Bael patra) that was procured from Bastar forest. Leaves from different plants were surface sterilized and were cut into small pieces of size 1 cm for the isolation of endophytes as per protocol of Rehman et al. [15].

### Production and partial purification of culture filtrates

The culture filtrates were produced by inoculating 5 mm of mycelial mass in 100 ml of Richard’s broth. The liquid cultures are then filtered through Whatman filter paper after incubation for 15 days at 26 °C, 120 rpm. The collected filtrate is then passed through 0.22 µm membrane filter to make it cell free [16].

The culture filtrates were then extracted with a polar solvent like ethyl acetate. The extracted solvents were then transferred to crucibles and were evaporated to dryness using Rotavapor. The remaining solid extracts are then dissolved in dimethylsulfoxide (5%) and stored at – 20 °C for further use.

### Antibacterial assays

The partially purified fungal extracts (100 µg/ml) were then evaluated for their antimicrobial activity for range of pathogenic microorganisms containing both Gram’s positive and Gram’s negative bacteria using a most common in vitro technique of agar well diffusion, which screens the activity at primary level efficiently.

#### Agar well diffusion assay

To screen the antibacterial activity of ethyl acetate residues from fungal filtrates, an agar well diffusion assay was performed. Initially, 5 mm wells were created in Mueller–Hinton agar (MHA, HiMedia) plates using an autoclaved cork borer. Each well was then filled with 30 µl of the test samples at a concentration of 100 µg/ml and allowed to diffuse for 15–20 min. Streptomycin (100 µg/ml) and DMSO (5%) served as positive and negative controls, respectively. Following diffusion, molten MHA was poured into the plates to seal the wells, and the plates were left undisturbed until the agar solidified. After solidification, the plates were inoculated with a 0.5 McFarland standard suspension of bacterial cultures and incubated at 37 °C. The antibacterial activity was assessed by measuring the zone of inhibition around each well, indicating the effectiveness of the test samples in preventing bacterial growth [17].

#### Minimal inhibitory concentration

Different concentrations of the ethyl acetate extract of positive fungi were also tested against an array of bacteria by broth micro dilution method. To achieve the level of 5 × 10^8^ cfu/ml, the turbidity of the bacterial cultures was set according to the standard McFarland solution (0.5 M). Further to reach the final concentration of cells around 1.5 × 10^6^ cfu/ml, the bacterial suspensions were diluted 100 times with sterile MH broth. The lowest concentration of the effective fungal residue that inhibits the growth of bacterial suspensions is referred as MIC. Streptomycin was used as the positive control, while DMSO (5%) served as the negative control. Each test was conducted in triplicate to ensure the reliability of the results, and the minimum inhibitory concentration (MIC) was determined as described by Kowalska-Krochmal and Dudek-Wicher [18].

### Identification of bioactive fungi

Genomic DNA from the bioactive fungi was isolated using the Wizard® Genomic DNA Purification Kit (Promega, Madison, USA) according to the manufacturer’s instructions. Internal transcribed spacer (ITS) regions ITS 1 and ITS 4 were amplified with an Applied Biosystems Thermocycler [19]. The PCR reaction mixture (20 µl) consisted of approximately 100 ng of fungal genomic DNA, 2 µl of dNTP mixture (2 mM), 2 µl of Taq buffer with MgCl_2_ (10X), 1 µl of each primer (ITS 1 and ITS 4, 10 pmol), and 1.5 U of Taq polymerase. The thermocycler was programmed with the following conditions: initial denaturation at 94 °C for 5 min, followed by 30 cycles of denaturation at 96 °C for 45 s, annealing at 58 °C for 90 s, extension at 72 °C for 90 s, and a final extension at 72 °C for 9 min. PCR amplification was confirmed using agarose gel electrophoresis. The purified amplicons were sequenced by Xcelris Lab, Ahmedabad, Gujarat, India. The sequencing results were analyzed using BLAST similarity software to identify the closest matching species. For phylogenetic analysis, ITS sequences from representative species of the *F. solani* species complex as detailed in Geiser et al. [20] were included. Maximum likelihood (ML) and maximum parsimony (MP) analyses for constructing the phylogenetic tree were performed using MEGAXv10.4 [21].

### GC/MS analysis of the bioactive compounds

The ethyl acetate extract of bioactive fungi was further analyzed for the constituent compounds using PerkinElmer Clarus 680 system (PerkinElmer, Inc. U.S.A). This instrument is equipped with fused nonpolar silica column of dimensions 30 m × 250 μm × 0.25 μm. The flow rate of helium gas was maintained at 1 ml/min that was used as carrier gas. The column oven temperature was heated up to 60 °C for 10 min then to 280–300 °C for 3 min. The compounds were identified on basis of retention indices with the compounds present in Willey and NIST libraries [22].

### In silico studies

#### Receptor preparation

The spike glycoprotein of SARS-CoV-2, which plays a key role in the virus’s pathogenicity, was chosen as the target for this study. The protein structure, identified by PDB ID: 6VXX and having a resolution of 2.80 Å, was obtained from the Research Collaboratory for Structural Bioinformatics Protein Data Bank (RCSB-PDB) [23]. To prepare the protein for docking studies, all heteroatoms and water molecules were removed from the structure.

#### Ligand’s preparation

A total of 45 ligands isolated from the endophytic fungi #3AMLBF were selected for virtual screening. The 3-D structures of these ligands were obtained in SDF format from the PubChem database and were manually curated to create a ligand library [24]. The physicochemical properties of the ligands were analyzed using online tools [25], and Lipinski’s rule of five was considered to evaluate their drug-likeness [26] (Table 1). Chloroquine, a known inhibitor of SARS-CoV, was used as a positive control for comparative studies [27].

**Table 1:** Physiochemical or ADME properties of ligands

S. No	Ligands	ADME properties (Lipinski’s rule of five)		Drug likeliness
		Properties	Values	
1	Cyclohexene, 1-methyl-4-(1-methylethenyl)	Molecular weight (< 500 Da)	170.68	Yes
		LogP (< 5)	3.3	
		H-bond donor (5)	0	
		H-bond acceptor (< 10)	0	
		Molar refractivity	45.9	
2	Cyclohexene,1-methyl-4-(1-methylethylidene)-	Molecular weight (< 500 Da)	136	Yes
		LogP (< 5)	2.8	
		H-bond donor (5)	0	
		H-bond acceptor (< 10)	0	
		Molar refractivity	45	
3	Aceticacid,2-ethylhexylester	Molecular weight (< 500 Da)	172	Yes
		LogP (< 5)	3	
		H-bond donor (5)	0	
		H-bond acceptor (< 10)	2	
		Molar refractivity	49	
4	Octane,3,4,5,6-tetramethyl-	Molecular weight (< 500 Da)	170	Yes
		LogP (< 5)	4.5	
		H-bond donor (5)	0	
		H-bond acceptor (< 10)	0	
		Molar refractivity	57.2	
5	2-ETHYL-1-HEXYLPROPIONATE	Molecular weight (< 500 Da)	186	Yes
		LogP (< 5)	3.1	
		H-bond donor (5)	0	
		H-bond acceptor (< 10)	2	
		Molar refractivity	54.5	
6	Octane,2,3,3-trimethyl-	Molecular weight (< 500 Da)	156	Yes
		LogP (< 5)	4.2	
		H-bond donor (5)	0 s	
		H-bond acceptor (< 10)	0	
		Molar refractivity	52.7	
7	Cyclohexasiloxane,dodecamethyl-	Molecular weight (< 500 Da)	44	Yes
		LogP (< 5)	4.4	
		H-bond donor (5)	0	
		H-bond acceptor (< 10)	6	
		Molar refractivity	89.9	
8	Octane,2,3,6,7-tetramethyl-	Molecular weight (< 500 Da)	170	Yes
		LogP (< 5)	4.3	
		H-bond donor (5)	0	
		H-bond acceptor (< 10)	0	
		Molar refractivity	57.2	
9	Bicyclo[7.2.0]Undec-4-ene,4,11,11-trimethyl-8-	Molecular weight (< 500 Da)	204	Yes
		LogP (< 5)	4.7	
		H-bond donor (5)	0	
		H-bond acceptor (< 10)	0	
		Molar refractivity	66.7	
10	1,3,6,10-Dodecatetraene,3,7,11-trimethyl-,(Z,E)-	Molecular weight (< 500 Da)	204	Yes
		LogP (< 5)	4.8	
		H-bond donor (5)	0	
		H-bond acceptor (< 10)	0	
		Molar refractivity	70.9	
11	2,6-DI-Butyl-2,5-cyclohexadiene-1,4-dione	Molecular weight (< 500 Da)	150	Yes
		LogP (< 5)	1.4	
		H-bond donor (5)	0	
		H-bond acceptor (< 10)	2	
		Molar refractivity	42.1	
12	Bis(2-ethylhexyl)ether	Molecular weight (< 500 Da)	242	Yes
		LogP (< 5)	5.4	
		H-bond donor (5)	0	
		H-bond acceptor (< 10)	1	
		Molar refractivity	77.4	
13	Azulene,1,2,3,5,6,7,8,8a-octahydro-1,4-dimethyl-7-(1-methyl	Molecular weight (< 500 Da)	128	Yes
		LogP (< 5)	2.4	
		H-bond donor (5)	0	
		H-bond acceptor (< 10)	0	
		Molar refractivity	43.1	
14	2-(4-Morpholinyl) ethanamine	Molecular weight (< 500 Da)	130	Yes
		LogP (< 5)	0.7	
		H-bond donor (5)	2	
		H-bond acceptor (< 10)	3	
		Molar refractivity	36.3	
15	Caryophyllene oxide	Molecular weight (< 500 Da)	220	Yes
		LogP (< 5)	3.9	
		H-bond donor (5)	0	
		H-bond acceptor (< 10)	1	
		Molar refractivity	66.2	
16	1,2-benzenedicarboxylicacid, diethylester	Molecular weight (< 500 Da)	222	Yes
		LogP (< 5)	2.0	
		H-bond donor (5)	0	
		H-bond acceptor (< 10)	4	
		Molar refractivity	58.3	
17	p-Sec-butylphenyl2,3-epoxypropylether	Molecular weight (< 500 Da)	206	Yes
		LogP (< 5)	2.7	
		H-bond donor (5)	0	
		H-bond acceptor (< 10)	2	
		Molar refractivity	60.7	
18	2,6-Bis(1,1-dimethylethyl)-4-(1-oxopropyl)phenol	Molecular weight (< 500 Da)	262	Yes
		LogP (< 5)	4.5	
		H-bond donor (5)	1	
		H-bond acceptor (< 10)	2	
		Molar refractivity	80.1	
19	1,6-Dioxacyclododecane-7,12-dione	Molecular weight (< 500 Da)	200	Yes
		LogP (< 5)	1.4	
		H-bond donor (5)	0	
		H-bond acceptor (< 10)	4	
		Molar refractivity	49.6	
20	1,1,4,7-tetramethyldecahydro-1 h-cycloprop	Molecular weight (< 500 Da)	222	Yes
		LogP (< 5)	3.4	
		H-bond donor (5)	1	
		H-bond acceptor (< 10)	1	
		Molar refractivity	65.9	
21	1,4-benzenediol,2,5-bis(1,1-dimethylethyl)-	Molecular weight (< 500 Da)	222	Yes
		LogP (< 5)	3.6	
		H-bond donor (5)	2	
		H-bond acceptor (< 10)	2	
		Molar refractivity	67.1	
22	1H-Indene-4-aceticacid,6-(1,1-dimethylethyl)-2,3-dihydro-1,	Molecular weight (< 500 Da)	260	Yes
		LogP (< 5)	3.8	
		H-bond donor (5)	1	
		H-bond acceptor (< 10)	77.8	
		Molar refractivity		
23	6-Undecylamine	Molecular weight (< 500 Da)	171	Yes
		LogP (< 5)	3.4	
		H-bond donor (5)	2	
		H-bond acceptor (< 10)	1	
		Molar refractivity	56.2	
24	3,5-di-tert-Butyl-4-hydroxyacetophenone	Molecular weight (< 500 Da)	248	Yes
		LogP (< 5)	4.1	
		H-bond donor (5)	1	
		H-bond acceptor (< 10)	2	
		Molar refractivity	7.5	
25	Formicacid,2,4,6-tri-t-butyl-phenylester	Molecular weight (< 500 Da)	290	Yes
		LogP (< 5)	5.1	
		H-bond donor (5)	0	
		H-bond acceptor (< 10)	2	
		Molar refractivity	89.2	
26	Benzene,1,3,5-tris(2,2-dimethylpropyl)-2-methyl-4,6-dinitro-	Molecular weight (< 500 Da)	392	Yes
		LogP (< 5)	5.0	
		H-bond donor (5)	0	
		H-bond acceptor (< 10)	4	
		Molar refractivity	113.9	
27	2,6-Bis(1,1-dimethylethyl)-4-(1-oxopropyl)phenol	Molecular weight (< 500 Da)	262	Yes
		LogP (< 5)	4.5	
		H-bond donor (5)	1	
		H-bond acceptor (< 10)	2	
		Molar refractivity	80.1	
28	4-Octadecylmorpholine	Molecular weight (< 500 Da)	339	Yes
		LogP (< 5)	6.2	
		H-bond donor (5)	0	
		H-bond acceptor (< 10)	1	
		Molar refractivity	124	
29	Hexadecanoicacid,methylester	Molecular weight (< 500 Da)	270	Yes
		LogP (< 5)	4.9	
		H-bond donor (5)	0	
		H-bond acceptor (< 10)	2	
		Molar refractivity	82.3	
30	2,3,4,5,6-Penta-O-acetyl-D-gluconitrile	Molecular weight (< 500 Da)	387	Yes
		LogP (< 5)	0.2	
		H-bond donor (5)	0	
		H-bond acceptor (< 10)	10	
		Molar refractivity	84.4	
31	Methyl2-acetamidoacrylate	Molecular weight (< 500 Da)	143	Yes
		LogP (< 5)	0.19	
		H-bond donor (5)	0	
		H-bond acceptor (< 10)	4	
		Molar refractivity	34.1	
32	1-Heptadecanamine,N,N-dimethyl-	Molecular weight (< 500 Da)	283	Yes
		LogP (< 5)	5.0	
		H-bond donor (5)	0	
		H-bond acceptor (< 10)	0	
		Molar refractivity	109	
33	3,4-Hexadienal,2-butyl-2-ethyl-5-methyl-	Molecular weight (< 500 Da)	194	Yes
		LogP (< 5)	3.8	
		H-bond donor (5)	0	
		H-bond acceptor (< 10)	1	
		Molar refractivity	61.2	
34	9,12-Octadecadienoicacid,methylester	Molecular weight (< 500 Da)	294	Yes
		LogP (< 5)	5.0	
		H-bond donor (5)	0	
		H-bond acceptor (< 10)	2	
		Molar refractivity	91.2	
35	Methyl9,9-dideutero-octadecanoate	Molecular weight (< 500 Da)	298	Yes
		LogP (< 5)	5.0	
		H-bond donor (5)	0	
		H-bond acceptor (< 10)	2	
		Molar refractivity	104.4	
36	Morpholine,4-octadecyl-	Molecular weight (< 500 Da)	339	Yes
		LogP (< 5)	6.0	
		H-bond donor (5)	0	
		H-bond acceptor (< 10)	1	
		Molar refractivity	124.1	
37	Octadecanoicacid,methylester	Molecular weight (< 500 Da)	298	Yes
		LogP (< 5)	5.6	
		H-bond donor (5)	0	
		H-bond acceptor (< 10)	2	
		Molar refractivity	104.4	
38	1H-Purin-6-amine, [(2-fluorophenyl)methyl]-	Molecular weight (< 500 Da)	243	Yes
		LogP (< 5)	2.1	
		H-bond donor (5)	2	
		H-bond acceptor (< 10)	4	
		Molar refractivity	65.4	
39	d-Glucitol,penta-O-acetyl-1-O-methyl-	Molecular weight (< 500 Da)	380	Yes
		LogP (< 5)	0.4	
		H-bond donor (5)	0	
		H-bond acceptor (< 10)	10	
		Molar refractivity	60.6	
40	Nonadecane	Molecular weight (< 500 Da)	228	Yes
		LogP (< 5)	0.4	
		H-bond donor (5)	0	
		H-bond acceptor (< 10)	0	
		Molar refractivity	50.0	
41	Di-n-octylphthalate	Molecular weight (< 500 Da)	352	Yes
		LogP (< 5)	0.6	
		H-bond donor (5)	0	
		H-bond acceptor (< 10)	4	
		Molar refractivity	69.6	
42	3-(2,2-Diphenylvinyl)-3-isobutyl-2-methylperhydropyrrolo[1,2	Molecular weight (< 500 Da)	372	Yes
		LogP (< 5)	0.2	
		H-bond donor (5)	0	
		H-bond acceptor (< 10)	3	
		Molar refractivity	94.2	
43	Squalene	Molecular weight (< 500 Da)	360	Yes
		LogP (< 5)	0	
		H-bond donor (5)	0	
		H-bond acceptor (< 10)	0	
		Molar refractivity	0.0	
44	6,11-Dimethyl-2,6,10-dodecatrien-1-ol	Molecular weight (< 500 Da)	184	Yes
		LogP (< 5)	1.02	
		H-bond donor (5)	0	
		H-bond acceptor (< 10)	1	
		Molar refractivity	49.4	
45	Anthraergosta-5,7,9,22-tetren-3-ol *p*-chlorobenzoate	Molecular weight (< 500 Da)	487	Yes
		LogP (< 5)	2.2	
		H-bond donor (5)	0	
		H-bond acceptor (< 10)	2	
		Molar refractivity	121.7	

#### Molecular docking of ligands with proteins

Molecular docking studies were conducted using AutoDock Vina to examine the interactions between the selected ligands and the target protein. Each ligand was first optimized using the universal force field (UFF) and then converted into.pdbqt format for docking. The docking process was performed as blind docking, with an exhaustiveness value set to 10. The interactions between the ligands and the protein were visualized using Discovery Studio Visualizer 4.0 [28].

#### Bioavailability radar

SwissADME is a free, web-based computational tool developed by the Swiss Institute of Bioinformatics to predict physicochemical properties, pharmacokinetics (ADME), drug-likeness, and medicinal chemistry friendliness of small molecules. The tool uses validated in silico models to estimate a compound’s suitability as an oral drug candidate.

## Results and discussion

### Results

#### Isolation of fungal endophytes

Around 16 different endophytes based on their morphological characters were isolated from leaves of *A. marmelos*. These isolated fungi were then screened for their antibacterial activities against both gram positive and gram negative bacteria.

#### In vitro antibacterial activities

Among 16 isolated fungi, only 5 fungi showed potential to inhibit this array of bacteria containing *S. aureus*, *Pseudomonas aeruginosa*, *Escherichia coli*. This was calculated using both zone of inhibition (ZOI) in mm and minimal inhibitory concentration (MIC) in µg/ml. The data are shown in Tables 2 and 3. Based on values of ZOI, these fungi were classified as weak (< 12 mm), moderate (12–16 mm), and strong (˃ 16 mm). #6 AMLBF and #11 AMLBF showed weak potential against *Staphylococcus* whereas #3 AMLBF and #12 AMLBF showed moderate results. Only two fungi (#3 AMLBF, #12 AMLBF) showed moderate activities against *Pseudomonas* whereas all 5 fungi showed moderate and strong potential against *E. coli*. Streptomycin inhibited all the three bacteria with strong potential much greater than the best isolated fungi #3 AMLBF. The MIC of isolated fungi showing potential ranged from 0.49–2.00 µg/ml. The ethyl acetate residue of #3 AMLBF showed MIC of 0.49 µg/ml against *Staphylococcus* and *Pseudomonas* whereas MIC of 0.77 µg/ml against *E. coli*.

**Table 2:** Zone of inhibition produced by ethyl acetate residues of isolated fungi against gram positive and gram negative bacteria

Culture code	Zone of Inhibition (mm)		
	*Staphylococcus aureus*,	*Psuedomonas aeruginosa*,	*Escherichia coli*
#1 AMLBF	**–**	**–**	**–**
#2 AMLBF	**–**	**–**	20.33 ± 1.53
#3 AMLBF	**12.17 ± 0.76**	**12.33 ± 0.58**	**21.67 ± 0.97**
#4 AMLBF	–	**–**	–
#5 AMLBF	–	**–**	–
#6 AMLBF	11.33 ± 0.15	–	16 ± 0.58
#7 AMLBF	–	**–**	–
#8 AMLBF	–	**–**	–
#9 AMLBF	–	**–**	–
#10 AMLBF	–	**–**	–
#11 AMLBF	11.67 ± 0.58	–	14.33 ± 0.43
#12 AMLBF	12.67 ± 0.58	11.67 ± 0.53	12.67 ± 0.38
#13 AMLBF	–	–	–
#14 AMLBF	–	–	–
#15 AMLBF	–	–	–
#16 AMLBF	–	–	–
Streptomycin	15.3 ± 0.6	17 ± 0.8	26 ± 1
DMSO (5%)	–	–	–

**Table 3:** Broth dilution assay of bioactive fungi against array of bacteria

Culture code	Minimal inhibitory concentration (µg/ml)		
	*S. aureus*,	*P. aeruginosa*,	*E. coli*
#2 AMLBF	–	–	0.8
#3 AMLBF	0.49	0.49	0.77
#6 AMLBF	0.89	–	1.88
#11 AMLBF	0.82	1.43	1.96
#12 AMLBF	0.5	0.6	2
Streptomycin	0.24	0.38	0.42
DMSO (5%)	–	–	–

#### Identification of potential fungi

**Fig. 1:**
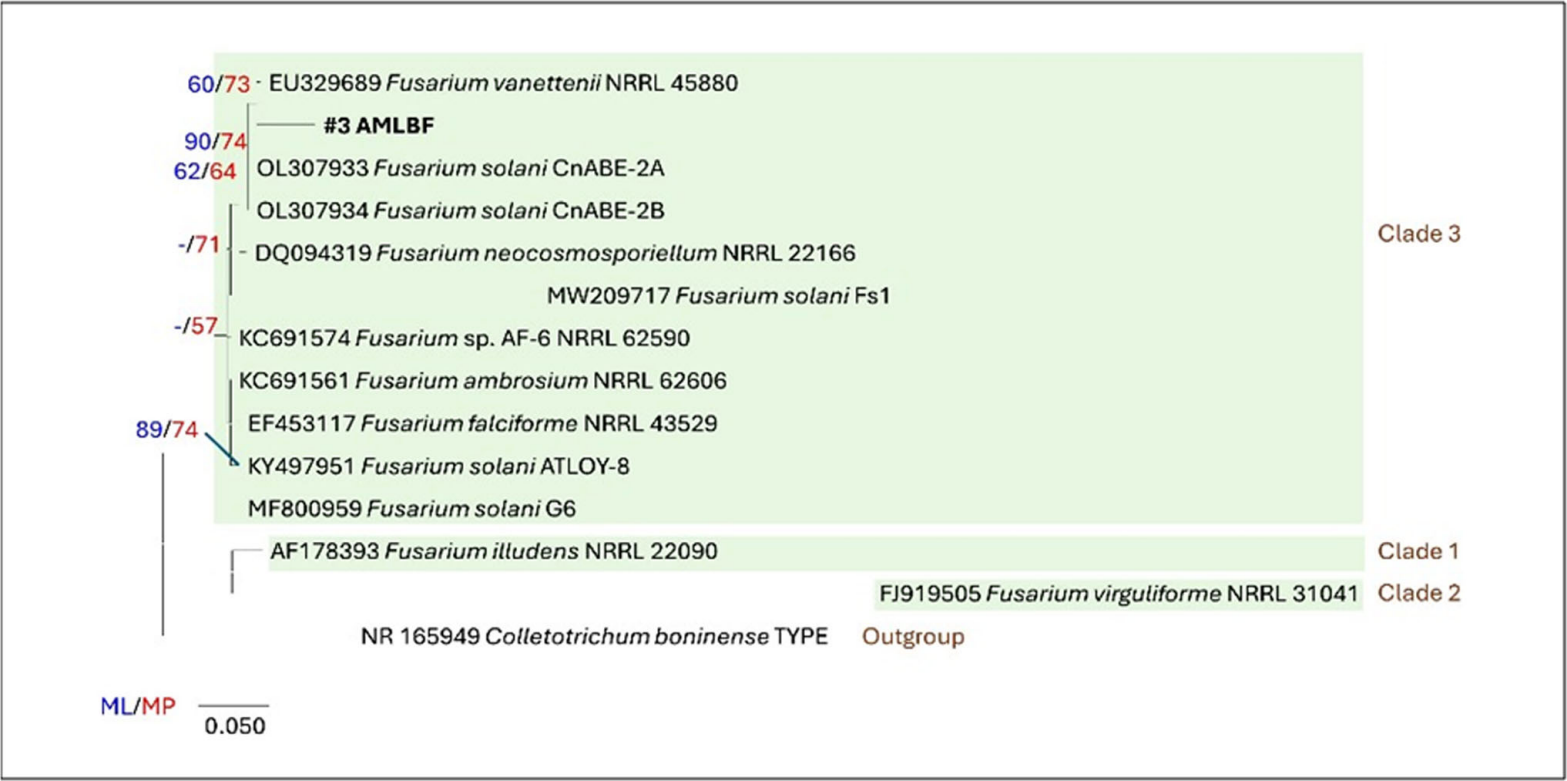
Maximum likelihood (ML) and maximum parsimony (MP) phylogenetic analysis of the bioactive fungi

To accurately identify the bioactive fungal isolate, an in-depth evolutionary analysis was conducted using a combination of maximum likelihood (ML) and maximum parsimony (MP) methods. This approach allows for a robust understanding of the genetic relationships and evolutionary history of the isolate, providing critical insights into its classification. The analysis was comprehensive, involving 14 nucleotide sequences, each carefully selected to ensure a representative dataset. To maintain the integrity of the analysis, only those positions with more than 95% site coverage were included. This criterion ensures that alignment gaps, missing data, and ambiguous bases were tolerated in less than 5% of positions, thereby focusing the analysis on the most reliable and informative parts of the sequences. This filtering resulted in a final dataset composed of 358 positions, providing a solid foundation for constructing phylogenetic trees. The MP tree, a fundamental component of this analysis, was constructed using the Tree-Bisection-Regrafting (TBR) algorithm, a powerful method for optimizing tree topology. The process began with the random addition of sequences, performed across 10 replicates to ensure variability and accuracy in the resulting tree structure. Meanwhile, the maximum likelihood analysis was executed using the Tamura 3-parameter model, which was identified as the best-fit model based on the data. This model, developed by Tamura in 1992, is well-regarded for its ability to accommodate differences in evolutionary rates across sites. The resulting phylogenetic tree, characterized by the highest log-likelihood value (− 1506.49), is visually presented in Fig. 1. To further validate the tree’s reliability, bootstrap values were calculated based on 100 replicates. These values, displayed alongside the branches, indicate the percentage of replicate trees in which the associated taxa consistently clustered together, providing a measure of confidence in the inferred relationships. The phylogenetic analysis conclusively placed the fungal isolate VM1 within Clade 3 of the *Fusarium solani* species complex (FSSC). More specifically, the isolate demonstrated a close genetic resemblance to *F. vanettenii* NRRL 45880, confirming its identity within this significant clade. This finding is pivotal, as it aligns the isolate with a well-characterized group of fungi known for their bioactive properties, laying the groundwork for further studies into its potential applications.

#### GC/MS analysis of the bioactive compound

The EA residue of bioactive fungi #3 AMLBF contained around 45 compounds which matched with the library having different retention time. Major compounds with maximum area were Cyclohexene, 1-methyl-4-(1-methylethenyl)-, 1,6-Dioxacyclododecane-7, 12-dione, 1H-Indene-4-aceticacid, 6-(1,1-dimethylethyl)-2,3-dihydro-1. These 45 compounds were further used for docking studies.

#### In silico anti-SARS-CoV activity

The in silico investigation of anti-SARS-CoV activity focused on evaluating the binding affinities between various ligands and the target SARS-CoV-2 *S*-glycoprotein, a critical protein responsible for viral entry into host cells. The binding affinities were systematically detailed in Table 4, highlighting the relative stability of the docked structures. The stability of these interactions is directly correlated with the binding affinity, with higher binding affinities indicating more stable ligand–protein complexes.

**Table 4:** Binding affinity of studied ligands with SARS-CoV-2 *S*-glycoprotein

	Ligands	Binding affinity
Control	Chloroquine	− 6.5
1	Cyclohexene,1-methyl-4-(1-methylethenyl)-	− 5.8
2	Cyclohexene,1-methyl-4-(1-methylethylidene)-	− 5.9
3	Aceticacid,2-ethylhexylester	− 4.6
4	Octane,3,4,5,6-tetramethyl-	− 5.4
5	2-Ethyl-1-hexylpropionate	− 5.2
6	Octane,2,3,3-trimethyl-	− 4.4
7	Cyclohexasiloxane,dodecamethyl-	− 2.8
8	Octane,2,3,6,7-tetramethyl-	− 5.0
9	Bicyclo[7.2.0]undec-4-ene,4,11,11-trimethyl-8-	− 6.4
10	1,3,6,10-Dodecatetraene,3,7,11-trimethyl-,(Z,E)-	− 5.3
11	2,6-Di-butyl-2,5-cyclohexadiene-1,4-dione	− 6.1
12	BIS(2-ETHYLHEXYL)ETHER	− 5.6
13	Azulene,1,2,3,5,6,7,8,8a-octahydro-1,4-dimethyl-7-(1-methyl	− 5.5
14	2-(4-Morpholinyl)ethanamine	− 4.3
15	**Caryophyllene oxide**	− **6.7**
16	1,2-Benzenedicarboxylicacid, diethylester	− 5.8
17	p-Sec-butylphenyl2,3-epoxypropylether	− 6.0
18	**2,6-Bis(1,1-dimethylethyl)-4-(1-oxopropyl)phenol**	− **6.7**
19	**1,6-Dioxacyclododecane-7,12-dione**	− **6.6**
20	**1,1,4,7-Tetramethyldecahydro-1 h-cycloprop**	− **7.1**
21	1,4-Benzenediol,2,5-bis(1,1-dimethylethyl)-	− 6.3
22	**1H-Indene-4-aceticacid,6-(1,1-dimethylethyl)-2,3-dihydro-1,**	− **7.5**
23	6-Undecylamine	− 4.9
24	**3,5-di-tert-Butyl-4-hydroxyacetophenone**	− **6.9**
25	Formicacid,2,4,6-tri-t-butyl-phenylester	− 6.0
26	Benzene,1,3,5-tris(2,2-dimethylpropyl)-2-methyl-4,6-dinitro-	− 5.9
27	2,6-Bis(1,1-dimethylethyl)-4-(1-oxopropyl)phenol	− 6.4
28	4-Octadecylmorpholine	− 5.0
29	Hexadecanoicacid,methylester	− 5.0
30	2,3,4,5,6-Penta-O-acetyl-D-gluconitrile	− 5.6
31	Methyl2-acetamidoacrylate	− 5.9
32	1-Heptadecanamine,N,N-dimethyl-	− 4.8
33	3,4-Hexadienal,2-butyl-2-ethyl-5-methyl-	− 5.0
34	9,12-Octadecadienoicacid,methylester	− 5.3
35	Methyl9,9-dideutero-octadecanoate	− 6.3
36	Morpholine,4-octadecyl-	− 4.3
37	Octadecanoicacid,methylester	− 4.4
38	**1H-Purin-6-amine, [(2-fluorophenyl)methyl]-**	− **7.2**
39	d-Glucitol,penta-O-acetyl-1-O-methyl-	− 5.9
40	Nonadecane	− 4.1
41	Di-n-octylphthalate	− 5.4
42	**3-(2,2-Diphenylvinyl)-3-isobutyl-2-methylperhydropyrrolo[1,2**	− **7.9**
43	Squalene	− 6.0
44	6,11-Dimethyl-2,6,10-dodecatrien-1-ol	− 5.1
45	**Anthraergosta-5,7,9,22-tetren-3-ol *p*-chlorobenzoate**	− **10.2**

*Ligands that have highest binding affinity are bold

Molecular docking analysis identified ligands that exhibited significant binding affinities, making them candidates for further detailed study. The target protein, consisting of three distinct chains—A, B, and C—played a crucial role in these interactions, as different ligands were observed to interact with various chains, contributing to the overall binding stability.

**Fig. 2:**
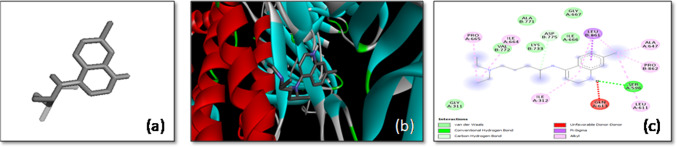
The molecular docking of SARS-CoV-2 Spike protein (S) with Chloroquine **a** 3-D structure of the ligand (Chloroquine), **b** Optimal binding configuration within the protein’s active site, and **c** Amino acid interactions between SARS-CoV-2 S protein and Chloroquine.

As a benchmark, the binding affinity of a control complex involving the *S*-glycoprotein and chloroquine, a well-known antimalarial drug that has been repurposed for SARS-CoV-2 research, was determined to be − 6.5 kcal/mol. This control interaction was mediated through a combination of hydrogen bonds and van der Waals forces, specifically involving residues such as Ser596 of the A-chain and Asp775 of the B-chain. In addition, alkyl and π-alkyl interactions were observed with several residues including Ile312, Ala647, Pro665, and Val772 from the A-chain, as well as Leu861 and Asp775 from the B-chain of the *S*-glycoprotein (Fig. 2). These interactions are consistent with previous studies, which have reported binding affinities of − 6.3 kcal/mol [27] and − 7.0 kcal/mol [12] for chloroquine when docked with the SARS-CoV-2 spike protein.

Out of the 45 ligands screened, those exhibiting binding affinities higher than that of chloroquine were selected for further analysis. Nine ligands emerged as promising candidates, demonstrating superior binding affinities compared to chloroquine. Among these, Anthraergosta-5,7,9,22-tetren-3-ol *p*-chlorobenzoate showed the highest binding affinity at − 10.2 kcal/mol, suggesting a potentially stronger and more stable interaction with the *S*-glycoprotein. The detailed interactions of these nine selected ligands are discussed further, providing insights into their potential as antiviral agents against SARS-CoV-2.

This comprehensive analysis highlights the potential of these ligands to disrupt the interaction between the SARS-CoV-2 *S*-glycoprotein and the ACE-2 receptor, which is essential for viral entry into host cells. The high binding affinities observed suggest that these compounds could serve as starting points for the development of new therapeutic agents aimed at treating COVID-19. Further experimental validation, including in vitro and in vivo studies, will be necessary to confirm the efficacy of these compounds as antiviral drugs.

The molecular docking analysis of Anthraergosta-5,7,9,22-tetren-3-ol *p*-chlorobenzoate with the SARS-CoV-2 spike (*S*) glycoprotein demonstrated a notable binding affinity of -10.2 kcal/mol, highlighting the ligand’s potential as a strong inhibitor of the virus’s interaction with host cells. This high binding affinity indicates a stable and energetically favorable interaction between the ligand and the target protein, which is crucial for inhibiting the virus’s ability to infect host cells. The docking study revealed that Anthraergosta-5,7,9,22-tetren-3-ol *p*-chlorobenzoate interacts with specific residues on both chain A and chain C of the *S*-glycoprotein. Notably, the ligand forms two hydrogen bonds with the Arg109 residues on both the A-chain and C-chain, establishing a strong anchoring interaction. These hydrogen bonds play a critical role in stabilizing the ligand within the protein’s binding site.

**Fig. 3:**
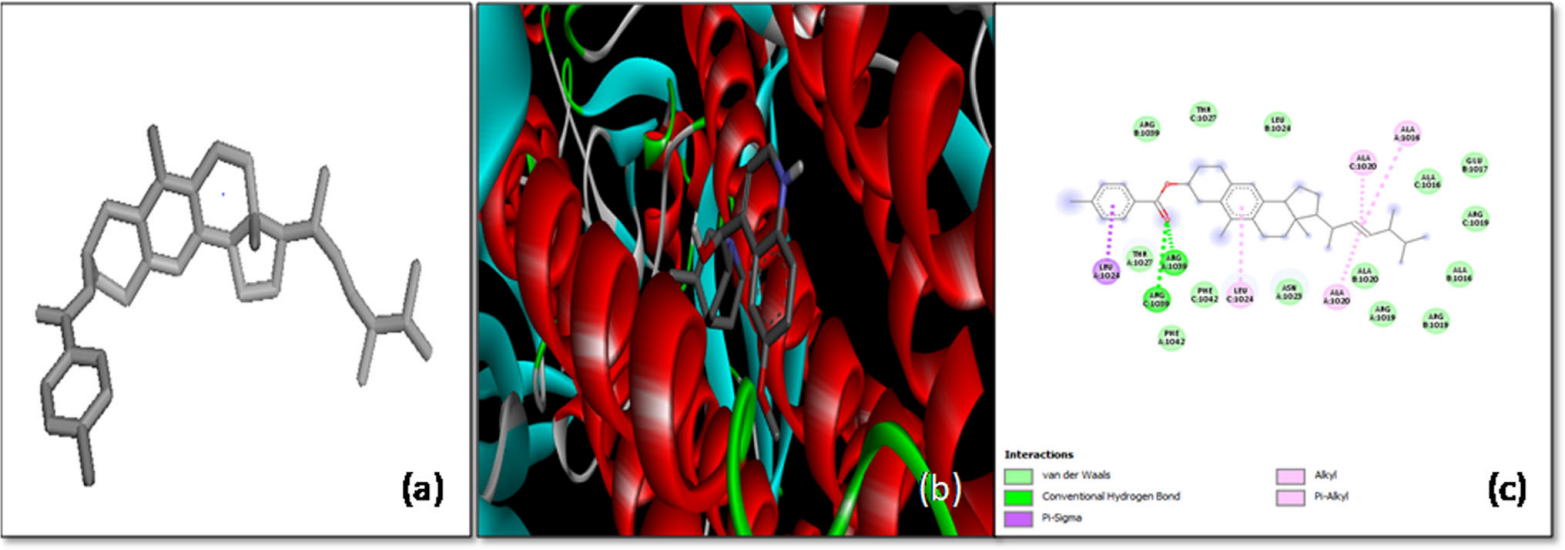
The molecular docking of SARS-CoV-2 Spike protein (S) with Anthraergosta-5,7,9,22-tetren-3-ol *p*-chlorobenzoate. **a** 3-D structure of the ligand (Anthraergosta-5,7,9,22-tetren-3-ol *p*-chlorobenzoate), **b** Optimal binding configuration within the protein’s active site, and **c** Amino acid interactions between SARS-CoV-2 S protein and Anthraergosta-5,7,9,22-tetren-3-ol *p*-chlorobenzoate

Additionally, the ligand engages in π-alkyl interactions with Ala1016 and Ala1020 residues of the A-chain, as well as Ala1020 and Leu1024 residues of the C-chain. These hydrophobic interactions further contribute to the stability of the ligand–protein complex, enhancing the likelihood of effective inhibition. Moreover, π-sigma interactions between the ligand and the Leu1024 residue of the A-chain provide an additional layer of stabilization (Fig. 3).

These findings suggest that Anthraergosta-5,7,9,22-tetren-3-ol *p*-chlorobenzoate may effectively block the *S*-glycoprotein’s interaction with the host cell’s ACE-2 receptor, thereby inhibiting viral entry. This compound emerges as a promising candidate for further experimental validation as an antiviral agent against SARS-CoV-2, offering potential therapeutic benefits in combating COVID-19.

**Fig. 4:**
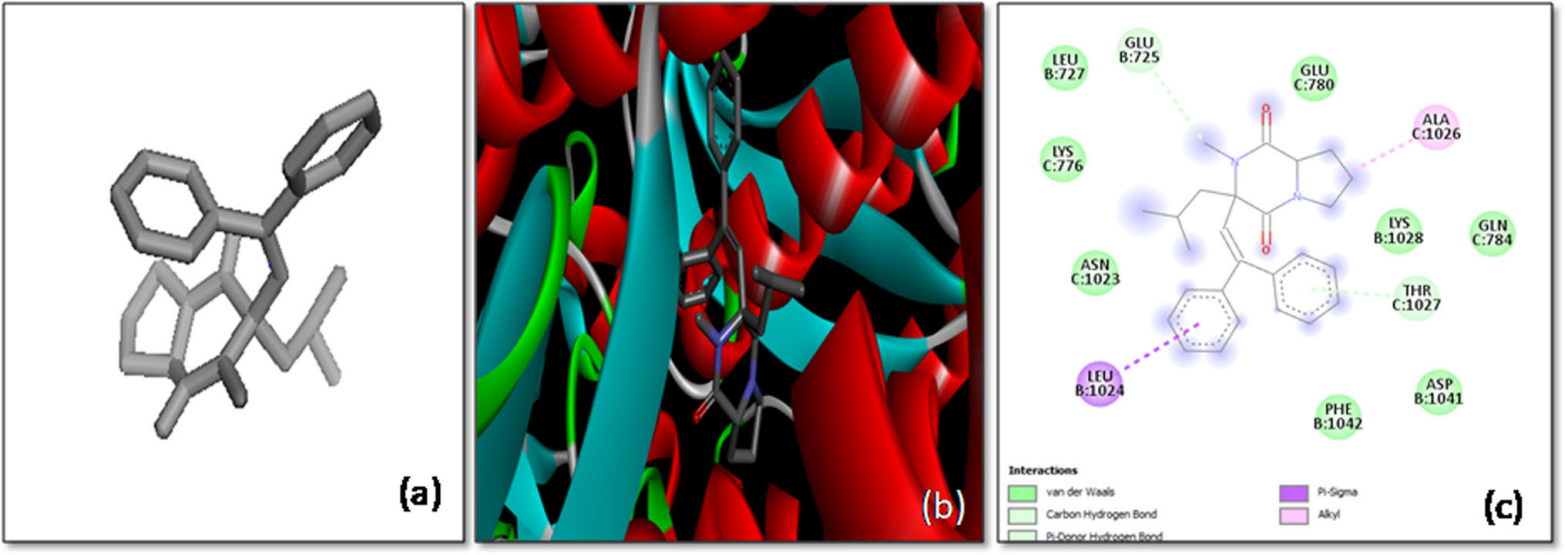
The molecular docking of SARS-CoV-2 *S*-glycoprotein with 3-(2,2-Diphenylvinyl)-3-isobutyl-2-methylperhydropyrrolo [1, 2]. **a** 3-D structure of the ligand, **b** optimal binding configuration within the protein’s active site, and **c** amino acid interactions between SARS-CoV-2 S protein and 3-(2,2-Diphenylvinyl)-3-isobutyl-2-methylperhydropyrrolo [1, 2]

The results of the molecular docking studies of various ligands with the SARS-CoV-2 spike (*S*) glycoprotein have provided valuable insights into potential antiviral candidates. The docking analysis of the compound 3-(2,2-Diphenylvinyl)-3-isobutyl-2-methylperhydropyrrolo [1,2-a] pyrazin-1,4-dione revealed a binding affinity of -7.9 kcal/mol, suggesting a relatively strong interaction with the target protein. This ligand primarily interacted with the B and C chains of the *S*-glycoprotein. The interactions included C-H bonding with the Glu725 residue of chain B and the Thr1025 residue of chain C, which likely contributed to the stability of the ligand–protein complex. Additionally, π-sigma interactions were observed with Leu1025 of chain B and Ala1026 of chain C, further stabilizing the complex (Fig. 4). These interactions highlight the ligand’s potential as a promising candidate for inhibiting the viral spike protein’s function, which is critical for viral entry into host cells.

**Fig. 5:**
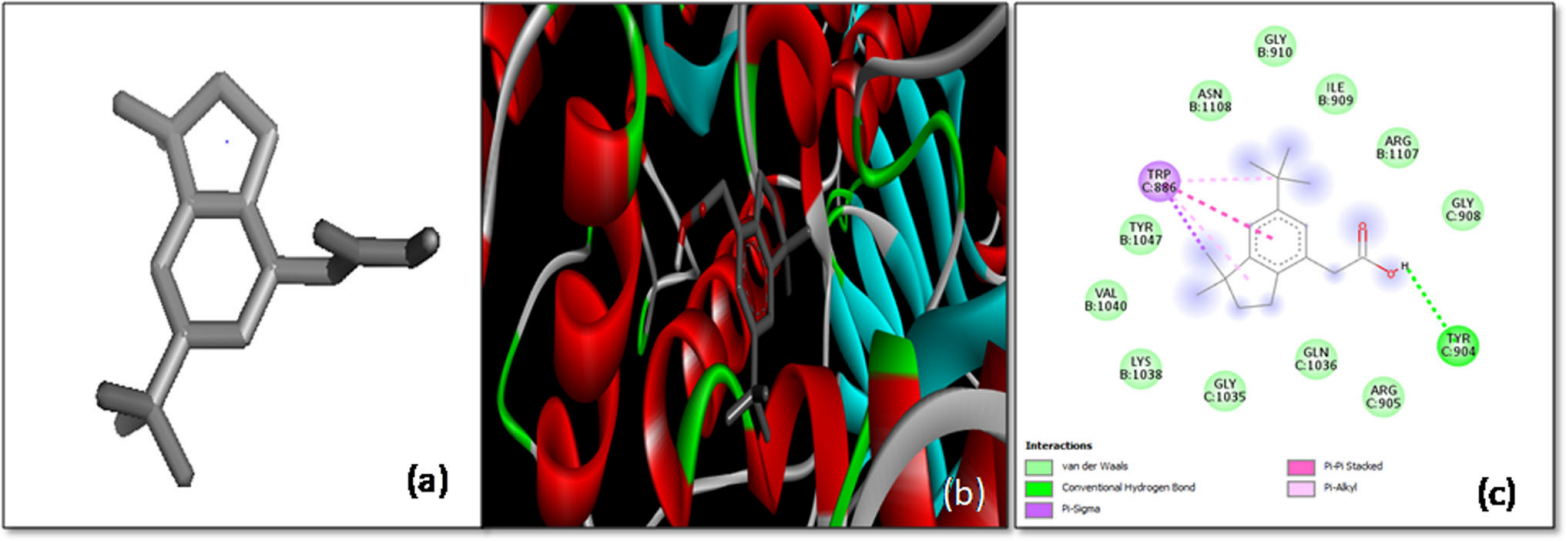
The molecular docking of SARS-CoV-2 *S*-glycoprotein with 1H-Indene-4-acetic acid, 6-(1,1-dimethylethyl)-2,3-dihydro-1. **a** 3-D structure of the ligand, **b** optimal binding configuration within the protein’s active site, and **c** amino acid interactions between SARS-CoV-2 S protein and 1H-Indene-4-acetic acid, 6-(1,1-dimethylethyl)-2,3-dihydro-1

Another significant finding emerged from the docking study of 1H-Indene-4-acetic acid with the *S*-glycoprotein, which revealed a binding affinity of − 7.5 kcal/mol. This ligand exhibited strong binding within the pocket of chain C of the spike protein. The interaction involved the formation of a hydrogen bond with the Tyr904 residue, a key interaction that enhances the ligand’s binding strength. Moreover, the ligand engaged in π-sigma, π-π stacked, and π-alkyl interactions with the Trp886 residue, contributing to the overall stability of the docked complex (Fig. 5). The combination of these interactions suggests that 1H-Indene-4-acetic acid may effectively hinder the spike protein’s ability to mediate viral entry, making it a potential antiviral agent.

**Fig. 6:**
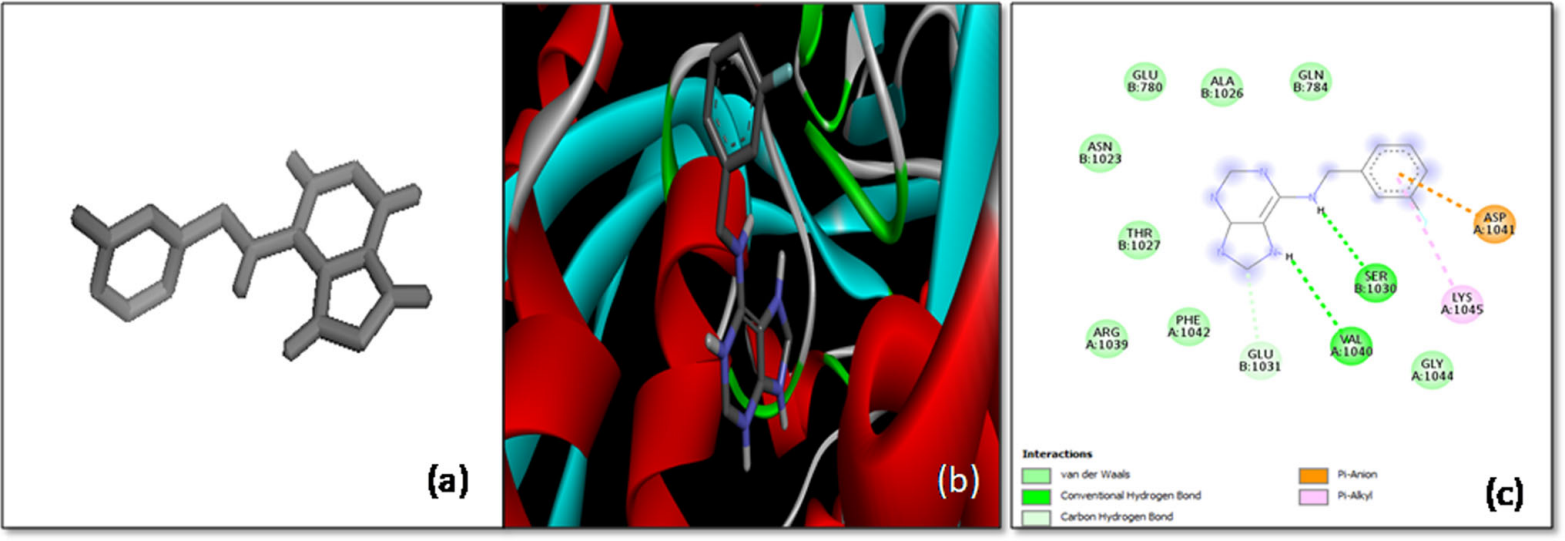
The molecular docking of SARS-CoV-2 *S*-glycoprotein with 1H-Purin-6-amine, [(2-fluorophenyl)methyl]. **a** 3-D structure of the ligand, **b** optimal binding configuration within the protein’s active site, and **c** amino acid interactions between SARS-CoV-2 S protein and 1H-Purin-6-amine, [(2-fluorophenyl)methyl]

The docking study of 1H-Purin-6-Amine, [(2-Fluorophenyl) Methyl] with the *S*-glycoprotein demonstrated a binding affinity of − 7.2 kcal/mol. This compound formed two critical hydrogen bonds with the Val1040 residue of chain A and the Ser1030 residue of chain B, indicating a strong interaction at these sites. Furthermore, the ligand interacted with Asp1041 and Lys1045 residues of chain A through π-anion and π-alkyl interactions, which are crucial for maintaining the stability of the ligand–protein complex (Fig. 6). These findings suggest that 1H-Purin-6-Amine could potentially inhibit the *S*-glycoprotein’s function, thereby blocking the virus’s ability to infect host cells.

**Fig. 7:**
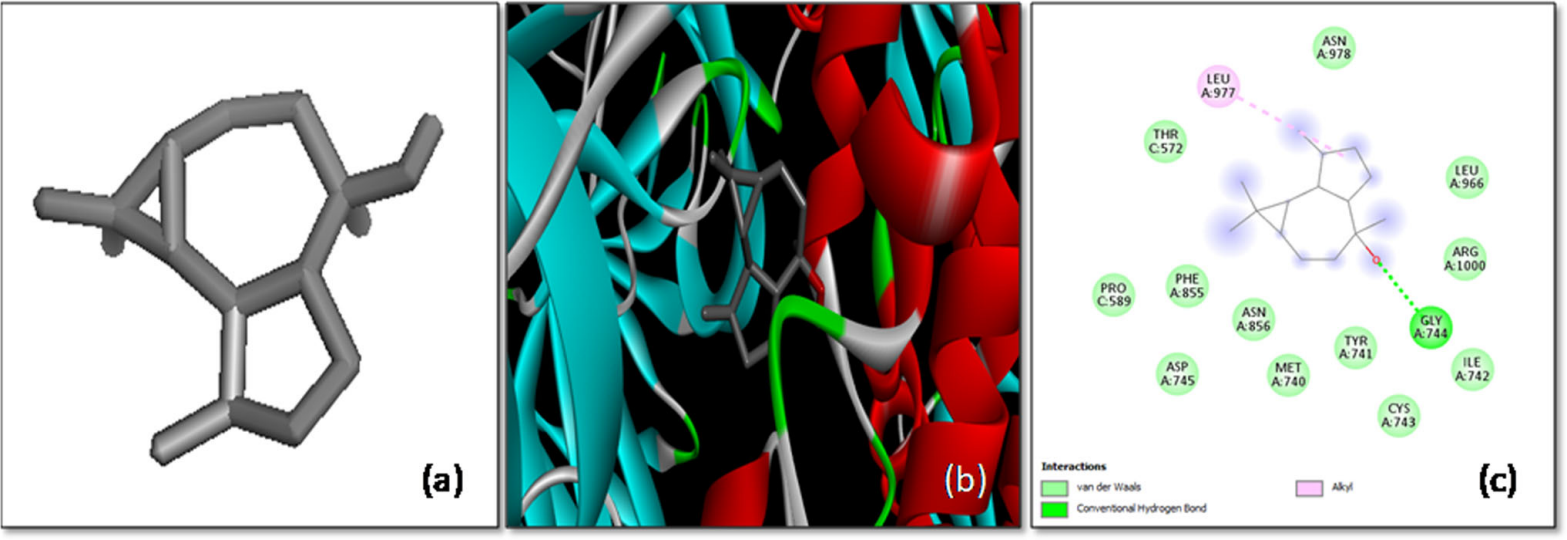
The molecular docking of SARS-CoV-2 *S*-glycoprotein with 1,1,4,7-Tetramethyldecahydro-1H-cyclopropaazulen-4-OL. **a** 3-D structure of the ligand, **b** optimal binding configuration within the protein’s active site, and **c** amino acid interactions between SARS-CoV-2 S protein and 1,1,4,7-Tetramethyldecahydro-1H-cyclopropaazulen-4-OL

The docking analysis of 1,1,4,7-Tetramethyldecahydro-1H-Cyclopropaazulen-4-OL with the *S*-glycoprotein showed a binding affinity of − 7.1 kcal/mol. This ligand predominantly interacted with chain A of the spike protein. A hydrogen bond was formed with the Gly744 residue, a significant interaction that helps anchor the ligand within the binding site. Additionally, alkyl interactions were observed with the Leu978 residue, further stabilizing the ligand–protein complex (Fig. 7). These interactions suggest that 1,1,4,7-Tetramethyldecahydro-1H-Cyclopropaazulen-4-OL could potentially serve as an effective inhibitor of the spike protein, thereby preventing viral entry.

These docking studies have identified several ligands with promising binding affinities and interactions with the SARS-CoV-2 *S*-glycoprotein. Each ligand demonstrated unique interactions that contribute to the overall stability and potential inhibitory effects on the viral spike protein. These findings lay the groundwork for further in vitro and in vivo studies to evaluate the antiviral efficacy of these compounds, with the ultimate goal of developing effective therapeutic agents against COVID-19.

The molecular docking studies revealed significant insights into the interactions of various ligands with the SARS-CoV-2 spike (*S*) glycoprotein, highlighting potential candidates for antiviral therapy. Out of the nine ligands investigated, four—3,5-di-tert-Butyl-4-hydroxyacetophenone, 2,6-Bis(1,1-dimethylethyl)-4-(1-oxopropyl) phenol, 1,6-Dioxacyclododecane-7,12-dione, and Caryophyllene oxide—demonstrated binding affinities comparable to the control drug, chloroquine. These affinities, ranging from − 6.6 to − 6.9 kcal/mol, suggest these ligands could effectively target the *S*-glycoprotein, potentially inhibiting the virus’s ability to enter host cells.

**Fig. 8:**
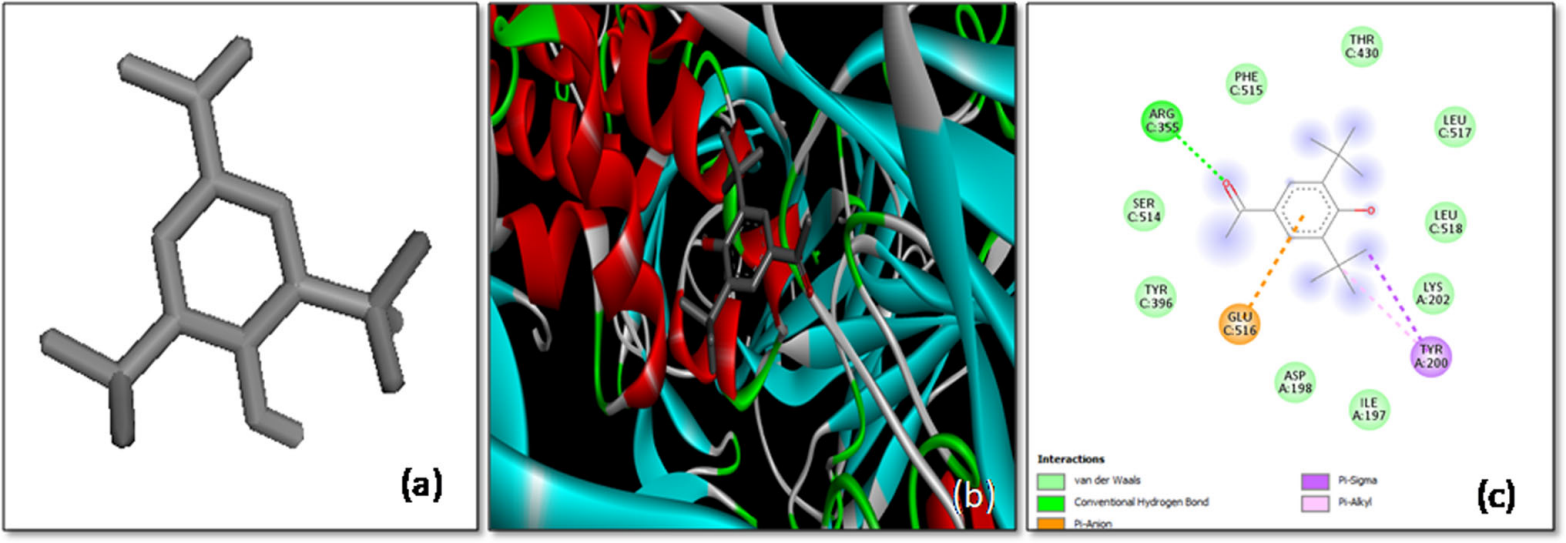
The molecular docking of SARS-CoV-2 *S*-glycoprotein and 3,5-di-tert-Butyl-4-hydroxyacetophenone. **a** 3-D structure of the ligand, **b** optimal binding mode within the protein pocket, and **c** amino acid interactions between SARS-CoV-2 S protein and 3,5-di-tert-Butyl-4-hydroxyacetophenone

**3,5-di-tert-Butyl-4-hydroxyacetophenone** exhibited a binding affinity of − 6.9 kcal/mol with the *S*-glycoprotein. This ligand formed a hydrogen bond with the Arg355 residue of chain A, which is crucial for its stable interaction within the protein’s binding pocket. The presence of a π-sigma interaction with the Tyr200 residue of chain A further enhanced the binding specificity, suggesting that this interaction might play a role in the ligand’s effectiveness as a potential inhibitor. Additionally, a π-anion interaction was observed with the Glu516 residue of chain C, indicating the ligand’s ability to establish multiple types of interactions within the protein structure. This diversity of interactions suggests that 3,5-di-tert-Butyl-4-hydroxyacetophenone may possess a well-rounded binding profile, making it a promising candidate for further study (Fig. 8).

**Fig. 9:**
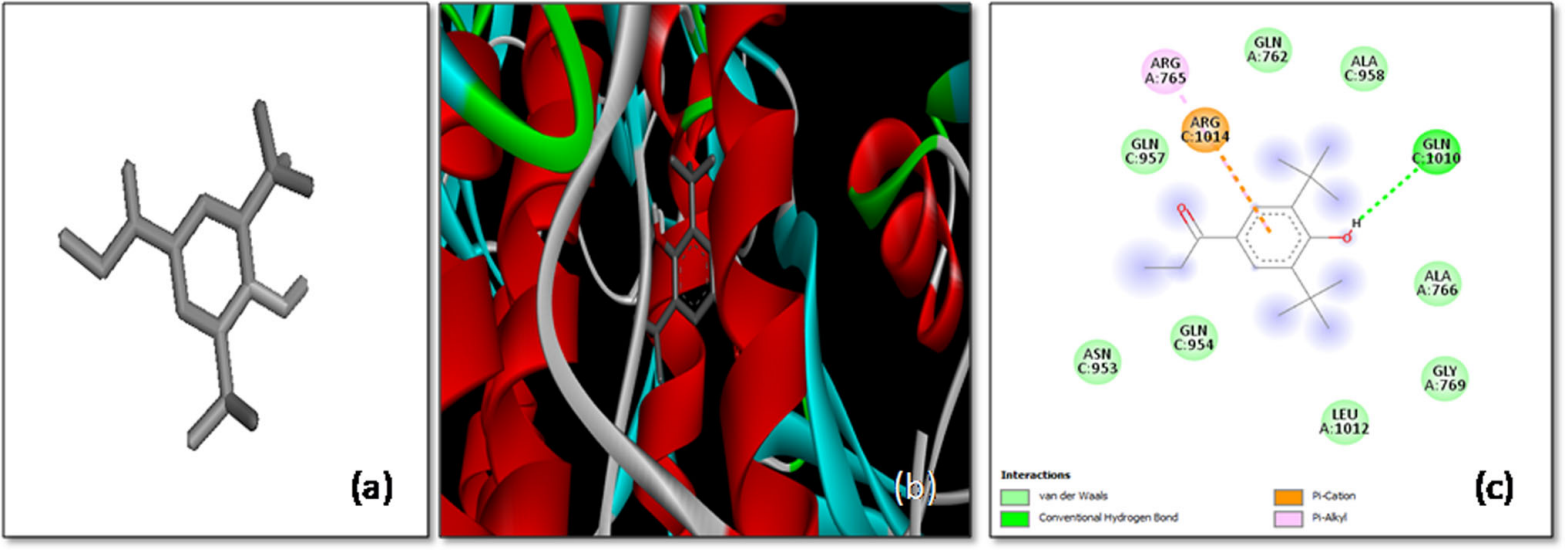
The molecular docking of SARS-CoV-2 *S*-glycoprotein and 2,6-Bis(1,1-dimethylethyl)-4-(1-oxopropyl) phenol. **a** 3-D structure of the ligand, **b** optimal binding mode within the protein pocket, and **c** amino acid interactions between SARS-CoV-2 S protein and 2,6-Bis(1,1-dimethylethyl)-4-(1-oxopropyl) phenol

**2,6-Bis(1,1-dimethylethyl)-4-(1-oxopropyl) phenol** also demonstrated a strong binding affinity of − 6.7 kcal/mol. The ligand’s ability to form a hydrogen bond with the Gly1010 residue of chain A played a significant role in stabilizing its position within the *S*-glycoprotein’s binding pocket. In addition to this hydrogen bond, the ligand engaged in π-alkyl and π-cation interactions with Arg765 and Ala1026 residues of chain A, as well as Arg1014 of chain C. These interactions suggest a balanced and stable binding profile, which could contribute to the ligand’s potential efficacy as an antiviral agent. The combination of hydrogen bonding and π-alkyl interactions indicates that this ligand may effectively inhibit the *S*-glycoprotein’s function, thereby preventing viral entry (Fig. 9).

**Fig. 10:**
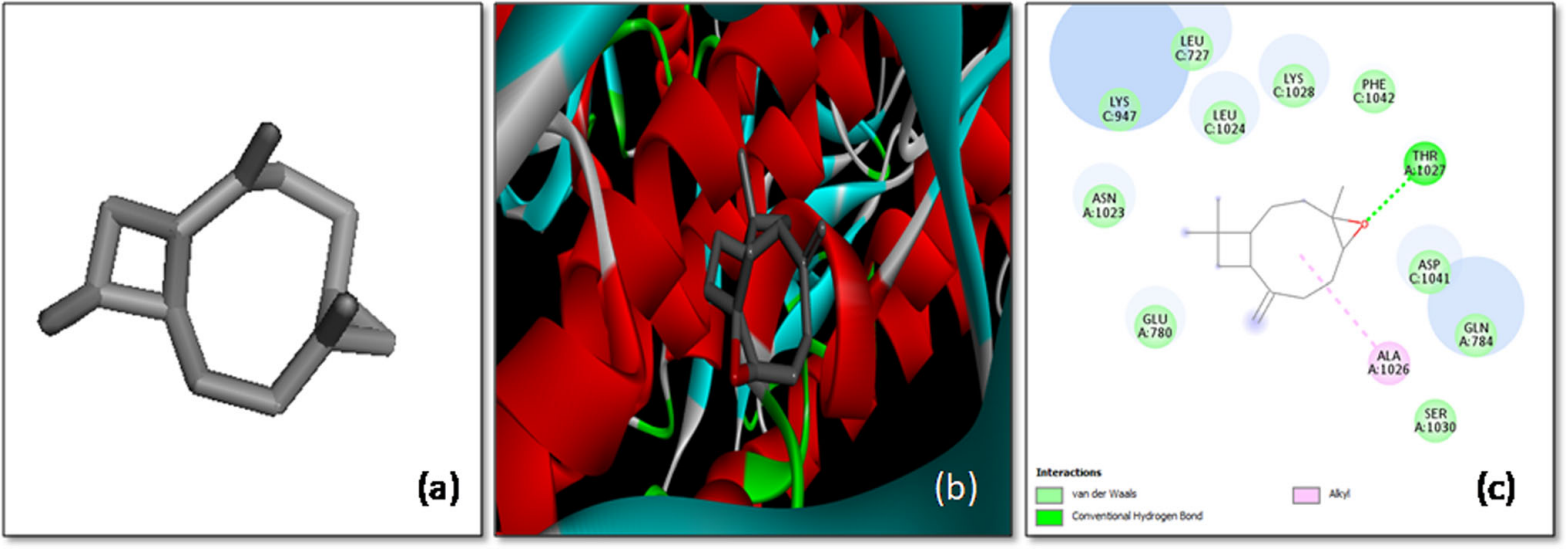
The molecular docking of SARS-CoV-2 *S*-glycoprotein and Caryophyllene oxide. **a** 3-D structure of the ligand, **b** optimal binding mode within the protein pocket, and **c** amino acid interactions between SARS-CoV-2 S protein and Caryophyllene oxide

**Caryophyllene oxide**, another promising candidate, showed a binding affinity of − 6.6 kcal/mol with the *S*-glycoprotein. This ligand formed a hydrogen bond with the Thr1027 residue of chain A, an interaction that is likely crucial for its binding stability. The ligand’s binding was further reinforced by π-alkyl and π-cation interactions, particularly with Arg765 of chain A and Arg1014 of chain C. These interactions not only contribute to the ligand’s stability within the protein pocket but also suggest that Caryophyllene oxide has the potential to disrupt the *S*-glycoprotein’s function. The strong and stable binding observed in this docking study highlights Caryophyllene oxide as a candidate worth further exploration for antiviral properties (Fig. 10).

**Fig. 11:**
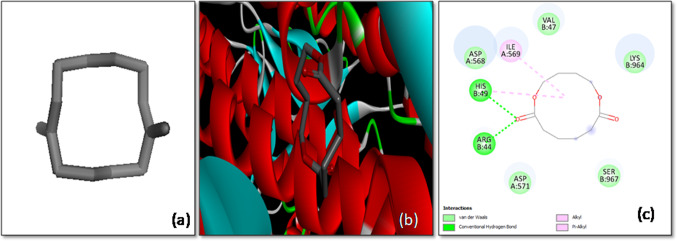
The molecular docking of SARS-CoV-2 *S*-glycoprotein and 1,6-Dioxacyclododecane-7,12-dione. **a** 3-D structure of the ligand, **b** optimal binding mode within the protein pocket, and **c** amino acid interactions between SARS-CoV-2 S protein and 1,6-Dioxacyclododecane-7,12-dione

**1,6-Dioxacyclododecane-7,12-dione** demonstrated a binding affinity of − 6.7 kcal/mol, making it another ligand with potential antiviral properties. This ligand established two hydrogen bonds with His49 and Arg44 residues of chain A, interactions that are likely critical for its binding affinity. The presence of these hydrogen bonds suggests that the ligand can effectively anchor itself within the *S*-glycoprotein’s binding pocket. Furthermore, an alkyl interaction with the Ile569 residue of chain B was noted, which may contribute to the ligand’s overall stability within the protein. The combination of hydrogen bonding and alkyl interactions indicates that 1,6-Dioxacyclododecane-7,12-dione has a robust binding profile, making it a promising candidate for further investigation (Fig. 11).

The molecular docking studies identified four ligands—3,5-di-tert-Butyl-4-hydroxyacetophenone, 2,6-Bis(1,1-dimethylethyl)-4-(1-oxopropyl) phenol, Caryophyllene oxide, and 1,6-Dioxacyclododecane-7,12-dione—that exhibited binding affinities comparable to chloroquine when docked with the SARS-CoV-2 *S*-glycoprotein. Each ligand demonstrated a unique interaction profile, involving a combination of hydrogen bonds, π-sigma, π-alkyl, and π-cation interactions, which contribute to their stability and potential effectiveness as inhibitors of the viral spike protein. These findings provide a strong foundation for further experimental validation and development of these ligands as potential therapeutic agents against COVID-19. The diversity in interaction types and the significant binding affinities suggest that these ligands could serve as promising candidates for drug development, offering hope for effective treatments in the fight against SARS-CoV-2.

#### Pharmacokinetics of potential ligands

**Fig. 12:**
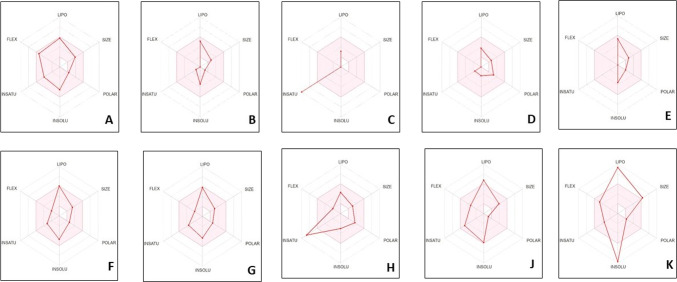
Radar diagrams **A** Chloroquine (Control), **B** Caryophyllene oxide, **C** 2,6-Bis(1,1-dimethylethyl)-4-(1-oxopropyl) phenol, **D** 1,6-Dioxacyclododecane-7,12-dione, **E** 1,1,4,7-Tetramethyldecahydro-1 h-cycloprop, **F** 1H-Indene-4-aceticacid,6-(1,1-dimethylethyl)-2,3-dihydro-1, **G** 3,5-di-tert-Butyl-4-hydroxyacetophenone, **H** 1H-Purin-6-amine, [(2-fluorophenyl) methyl, **J** 3-(2,2-Diphenylvinyl)-3-isobutyl-2-methyl per hydropyrrolo [1, 2], **K** Anthraergosta-5,7,9,22-tetren-3-ol *p*-chlorobenzoate

The Swiss ADME bioavailability radar illustrates the physicochemical suitability of the compound for oral administration, with the optimal drug-like space represented by the pink area (Fig. 12).

Among the evaluated phytochemicals, Anthraergosta-5,7,9,22-tetren-3-ol *p*-chlorobenzoate was selected as the lead compound based on its overall superior in silico pharmacokinetic and drug-likeness profile. The compound demonstrated high gastrointestinal absorption, comparable to chloroquine and all tested phytochemicals, indicating favorable oral uptake. Notably, it was predicted to be nonsubstrate for *P*-glycoprotein, suggesting reduced efflux liability and improved intracellular retention. In addition, the compound did not exhibit any inhibitory activity against CYP1A2 or CYP3A4, highlighting a lower risk of metabolic drug–drug interactions relative to reference drug chloroquine.

Although the compound displayed moderate aqueous solubility (ESOL Log *S* =  − 4.75), this limitation is offset by its high bioavailability score (0.85), one of the highest among the screened phytochemicals, indicating strong oral drug-likeness. Its moderate lipophilicity (iLOGP = 4.17) further supports an optimal balance between membrane permeability and solubility, avoiding the excessive lipophilicity observed in less favorable candidates as shown in Table 5. In contrast, other compounds with comparable or higher bioavailability exhibited undesirable pharmacokinetic liabilities, including *P*-gp substrate behavior, cytochrome P450 inhibition, or suboptimal lipophilicity.

**Table 5:** Pharmacokinetics of studied phytochemicals

	Phytochemicals	ESOL (Log S)	GIA	BBB permeant	*P*-gp substrate	CYP1A2 inhibitor	CYP3A4 inhibitor	(iLOGP)	Bioavailability score
Control	Chloroquine	− 4.55	High	Yes	No	Yes	Yes	4.15	0.55
1	Caryophyllene oxide	− 3.45	High	Yes	No	No	No	3.68	0.55
2	2,6-Bis(1,1-dimethylethyl)-4-(1-oxopropyl)phenol	− 5.56	High	No	Yes	No	No	3.54	0.55
3	1,6-Dioxacyclododecane-7,12-dione	− 1.76	High	Yes	No	No	No	1.53	0.55
4	1,1,4,7-Tetramethyldecahydro-1 h-cycloprop	-3.57	High	Yes	No	No	No	3.42	0.55
5	1H-Indene-4-aceticacid,6-(1,1-dimethylethyl)-2,3-dihydro-1,	− 3.72	High	No	Yes	No	No	8.77	0.87
6	3,5-di-tert-Butyl-4-hydroxyacetophenone	4.32	High	Yes	No	Yes	No	3.90	0.55
7	1H-Purin-6-amine, [(2-fluorophenyl)methyl]-	− 3.03	High	Yes	No	No	No	4.17	0.55
8	3-(2,2-Diphenylvinyl)-3-isobutyl-2-methylperhydropyrrolo[1,2	− 5.85	High	Yes	No	No	No	5.18	0.55
9	Anthraergosta-5,7,9,22-tetren-3-ol *p*-chlorobenzoate	− 4.75	High	Yes	No	No	No	4.17	0.85

### Discussion

The emergence of drug-resistant microbial strains and the continuous evolution of new pathogenic variants pose significant challenges to modern medicine. The relentless search for novel and effective treatments has prompted researchers to explore diverse natural sources, including traditional medicinal plants renowned for their therapeutic properties. These plants often provide a rich source of bioactive compounds with potential therapeutic benefits. However, harvesting these plants from their natural habitats can threaten biodiversity and is labor-intensive. An alternative approach is to utilize endophytes—microorganisms living within plant tissues without causing harm to their hosts. Endophytes have been shown to produce a range of bioactive compounds similar to those found in their host plants, making them a promising source of new pharmaceuticals.

In this study, we isolated endophytic fungi from the Indian medicinal plant *A. marmelos*, which grows in the unexplored forests of Bastar, a region affected by communal disturbances. *A. marmelos*, commonly known as bael, holds significant cultural and medicinal value in India. The plant’s leaves are known to produce myristic acid and linolenic acid, which contribute to its antimicrobial properties [29]. Our study underscores the potential of endophytes from *A. marmelos* as sources of novel antimicrobial agents, as these fungi often mirror the therapeutic properties of their host plants.

Historically, the Western Ghats have been a source of several notable endophytes. For instance, endophytic fungi such as *Alternaria* and *Curvularia* from *A. marmelos* have demonstrated significant antimicrobial activity against various bacterial strains [30]. The exploration of endophytes from the Bastar forests, including our earlier work on endophytes from *Curcuma caesia* [16], has been relatively sparse. This study extends this exploration to uncover the potential of endophytes from *A. marmelos* in combating pathogenic microbes.

Among the endophytic fungi studied, *F. vanettenii* emerged as a notable candidate with both antibacterial and antiviral properties. Known for its production of diverse secondary metabolites [31], *F. vanettenii* was investigated for its ability to produce bioactive compounds with potential therapeutic effects. Through gas chromatography–mass spectrometry (GC–MS) analysis, we identified 45 different compounds in the ethyl acetate extract of *F. vanettenii*. These compounds were subsequently evaluated for their potential to interact with the SARS-CoV-2 spike (*S*) glycoprotein, a crucial target for antiviral drug development.

The COVID-19 pandemic has highlighted the urgent need for effective therapies that are both potent and safe. The spike (*S*) glycoprotein of SARS-CoV-2 is central to the virus’s entry into host cells via the ACE-2 receptor [32]. Thus, targeting this protein offers a strategic approach to inhibit viral infection. Computational methods, such as molecular docking and virtual screening, have become essential tools in drug discovery. These techniques provide a cost-effective and time-efficient means to identify and optimize potential drug candidates. Previous studies have demonstrated the effectiveness of such computational approaches in predicting interactions between lead molecules and viral targets [10].

In our study, molecular docking was employed to assess the binding affinity of the 45 compounds identified from *F. vanettenii* with the SARS-CoV-2 *S*-glycoprotein. The docking results revealed several ligands with promising binding affinities, surpassing the control compound, chloroquine [11]. Among these ligands, nine exhibited particularly high binding affinities: 1H-Indene-4-acetic acid, 6-(1,1-dimethylethyl)-2,3-dihydro-1, 3,5-di-tert-Butyl-4-hydroxyacetophenone, anthraergosta-5,7,9,22-tetren-3-ol *p*-chlorobenzoate, 1H-Purin-6-Amine, [(2-Fluorophenyl)Methyl], 3-(2,2-Diphenylvinyl)-3-isobutyl-2-methylperhydro pyrrolo, 2,6-Bis(1,1-dimethylethyl)-4-(1-oxopropyl) phenol, Caryophyllene oxide, and Dioxacyclododecane-7,12-dione. These compounds demonstrated higher binding affinities for the SARS-CoV-2 *S*-glycoprotein compared to chloroquine, suggesting their potential as effective inhibitors [26, 33].

The binding affinities of these ligands indicate their potential to interfere with the virus’s ability to attach to host cells. For instance, the high binding affinities observed in this study imply that these compounds could potentially disrupt the interaction between the *S*-glycoprotein and the ACE-2 receptor, thereby impeding the viral entry process. Further, pharmacokinetic attributes confirm Anthraergosta-5,7,9,22-tetren-3-ol *p*-chlorobenzoate as the most promising lead candidate for further in vitro and in vivo validation, as well as structure-based optimization.

This compound serves as promising candidates for the development of new antiviral drugs targeting COVID-19 [34, 35].

The findings of this study emphasize the value of endophytes as untapped reservoirs of bioactive compounds with potential therapeutic applications. By leveraging the unique biosynthetic capabilities of endophytes like *F. vanettenii*, researchers can uncover novel compounds that may offer new avenues for treating drug-resistant infections and emerging viral diseases. The integration of computational methods with natural product research presents a powerful strategy for drug discovery, facilitating the identification of promising candidates for further validation [36].

This study highlights the potential of endophytic fungi from *A. marmelos* as sources of novel antimicrobial and antiviral agents. The successful identification of compounds with high binding affinities for the SARS-CoV-2 *S*-glycoprotein underscores the importance of exploring natural sources for drug discovery [37–39]. Future research should focus on validating the antiviral activity of these compounds through in vitro and in vivo studies, which will be crucial for assessing their efficacy and safety as potential treatments for COVID-19. If successful, these compounds could contribute significantly to the development of new therapeutic options and enhance our ability to combat both microbial resistance and viral infections.

## Conclusion

The rise of drug resistance has emerged as a significant challenge in treating common diseases, compounded by the appearance of new mutant variants resistant to standard antimicrobial therapies. In this context, the present study highlights the antimicrobial and antiviral potential of endophytic fungi isolated from the leaves of the sacred plant *A. marmelos*. The COVID-19 pandemic presented an unprecedented challenge to the global scientific community, underscoring the urgent need for effective antiviral drugs to combat life-threatening infections like SARS-CoV-2. Molecular docking has proved to be a valuable tool in the search for new therapeutic agents, offering insights into potential drug-target interactions. In this study, #3 AMLBF, an extract derived from *A. marmelos* endophytic fungi, has shown promise as a therapeutic candidate against SARS-CoV-2, with the potential for minimal side effects. The molecular docking of 45 compounds from #3 AMLBF identified several promising interactions with the viral *S*-glycoprotein, a critical protein involved in the virus’s entry into host cells. Among these, nine compounds were identified as potential inhibitors that could disrupt the interaction between the SARS-CoV-2 *S*-glycoprotein and the host ACE-2 receptor, a key mechanism in preventing viral infection. While these findings are promising, further research is needed to validate these compounds’ efficacy through in vitro and in vivo studies. If successful, these compounds could be developed into effective antiviral drugs, offering a new line of defense against COVID-19 and other viral infections.

## Data Availability

Data sharing is not applicable to this article as no datasets were generated or analyzed during the current study.
